# Coral-algae metabolism and diurnal changes in the CO_2_-carbonate system of bulk sea water

**DOI:** 10.7717/peerj.378

**Published:** 2014-05-22

**Authors:** Paul L. Jokiel, Christopher P. Jury, Ku’ulei S. Rodgers

**Affiliations:** Hawaii Institute of Marine Biology, University of Hawaii, Kaneohe, HI, United States

**Keywords:** Phase lag, Boundary layers, Coral, Algae, Coral reef, Aragnite saturation state, Photosynthesis, Calcification, Proton flux

## Abstract

Precise measurements were conducted in continuous flow seawater mesocosms located in full sunlight that compared metabolic response of coral, coral-macroalgae and macroalgae systems over a diurnal cycle. Irradiance controlled net photosynthesis (*P*_net_), which in turn drove net calcification (*G*_net_), and altered pH. *P*_net_ exerted the dominant control on [CO_3_^2−^] and aragonite saturation state (Ω_arag_) over the diel cycle. Dark calcification rate decreased after sunset, reaching zero near midnight followed by an increasing rate that peaked at 03:00 h. Changes in Ω_arag_ and pH lagged behind *G*_net_ throughout the daily cycle by two or more hours. The flux rate *P*_net_ was the primary driver of calcification. Daytime coral metabolism rapidly removes dissolved inorganic carbon (DIC) from the bulk seawater and photosynthesis provides the energy that drives *G*_net_ while increasing the bulk water pH. These relationships result in a correlation between *G*_net_ and Ω_arag_, with Ω_arag_ as the dependent variable. High rates of H^+^ efflux continued for several hours following mid-day peak *G*_net_ suggesting that corals have difficulty in shedding waste protons as described by the Proton Flux Hypothesis. DIC flux (uptake) followed *P*_net_ and *G*_net_ and dropped off rapidly following peak *P*_net_ and peak *G*_net_ indicating that corals can cope more effectively with the problem of limited DIC supply compared to the problem of eliminating H^+^. Over a 24 h period the plot of total alkalinity (*A_T_*) versus DIC as well as the plot of *G*_net_ versus Ω_arag_ revealed a circular hysteresis pattern over the diel cycle in the coral and coral-algae mesocosms, but not the macroalgae mesocosm. Presence of macroalgae did not change *G*_net_ of the corals, but altered the relationship between Ω_arag_ and *G*_net_. Predictive models of how future global changes will effect coral growth that are based on oceanic Ω_arag_ must include the influence of future localized *P*_net_ on *G*_net_ and changes in rate of reef carbonate dissolution. The correlation between Ω_arag_ and *G*_net_ over the diel cycle is simply the response of the CO_2_-carbonate system to increased pH as photosynthesis shifts the equilibria and increases the [CO_3_^2−^] relative to the other DIC components of [HCO_3_^−^] and [CO_2_]. Therefore Ω_arag_ closely tracked pH as an effect of changes in *P*_net_, which also drove changes in *G*_net_. Measurements of DIC flux and H^+^ flux are far more useful than concentrations in describing coral metabolism dynamics. Coral reefs are systems that exist in constant disequilibrium with the water column.

## Introduction

Recent field experiments have identified the need for accurate metabolic measurements on coral reefs at short time intervals in order to detect subtle aspects such as phase lags between the concentrations and flux rates of major metabolic parameters over the diurnal cycle (e.g., Shamberger et al., 2011; McMahon et al., 2013). There is a need to test the assumption that night calcification is very low and constant and that calcification is limited by inorganic carbon concentration in the form of CO_3_^2−^. Finely detailed diurnal metabolic measurements are difficult to obtain in field investigations. For example, Falter et al. (2012) based their extensive study at Nigaloo Reef, NW Australia on a total of 13 summer measurements of calcification taken at various times over 12 days between 08:00 and 18:00 with one night measurement at 21:00. The following winter a total of 11 more measurements were made over 4 days with no night measurements. These data are valuable, but do not provide the diurnal resolution that is needed to fully describe coral and coral reef metabolism. Thus we undertook a laboratory investigation that would provide such data.

## Background Information

The term Ω_arag_ is defined as:

(1) \begin{eqnarray*} \displaystyle {\Omega }_{\mathrm{arag}}=\frac{[{\mathrm{Ca}}^{2+}][{\mathrm{CO}}_{3}^{2-}]}{{K}_{s p}}&&\displaystyle \end{eqnarray*}

where *K_sp_* is the solubility constant of aragonite. The [Ca^2+^] in normal present-day oceanic seawater is essentially constant at 10.3 mmol kg^−1^ SW, normalized to salinity. Likewise, *K_sp_* is a constant (at a given temperature, pressure, and salinity), so in shallow oceanic waters Ω_arag_ is directly proportional to [CO_3_^2−^]. Changes in seawater pH shift the equilibria between the various forms of dissolved inorganic carbon (DIC) as follows:

(2) \begin{eqnarray*} \displaystyle {\mathrm{CO}}_{2}+{\mathrm{H}}_{2}\mathrm{O}\Leftrightarrow {\mathrm{H}}^{+}+{\mathrm{HCO}}_{3}^{-}\Leftrightarrow 2{\mathrm{H}}^{+}+{\mathrm{CO}}_{3}^{2-}.&&\displaystyle \end{eqnarray*}

Calcification inevitably produces an excess of H^+^ and thus reduces total alkalinity (*A_T_*) by two moles for every mole of CaCO_3_ precipitated (Kinsey, 1978; Smith & Kinsey, 1978). The correct equations for calcification are as follows:

(3) \begin{eqnarray*} \displaystyle {\mathrm{Ca}}^{2+}+({\mathrm{CO}}_{2}+{\mathrm{H}}_{2}\mathrm{O})\Leftrightarrow {\mathrm{CaCO}}_{3}+2{\mathrm{H}}^{+}&&\displaystyle \end{eqnarray*}

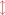

(4) \begin{eqnarray*} \displaystyle {\mathrm{Ca}}^{2+}+({\mathrm{H}}^{+}+{\mathrm{HCO}}_{3}^{-})\Leftrightarrow {\mathrm{CaCO}}_{3}+2{\mathrm{H}}^{+}&&\displaystyle \end{eqnarray*}

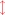

(5) \begin{eqnarray*} \displaystyle {\mathrm{Ca}}^{2+}+(2{\mathrm{H}}^{+}+{\mathrm{CO}}_{3}^{2-})\Leftrightarrow {\mathrm{CaCO}}_{3}+2{\mathrm{H}}^{+}&&\displaystyle \end{eqnarray*}

Equations 3–5 are written in two dimensions with a red arrow showing the relationship between the carbonate species (in parentheses) that shift with the changes in [H^+^] described as Eq. (2). Dissolution is the reverse of the calcification reaction. Net calcification (*G*_net_) is the sum of calcification (positive flux) and dissolution (negative flux). When the equations are written correctly in this manner the importance of protons becomes apparent with two moles of H^+^ produced for every mole of CaCO_3_ precipitated regardless of which form of dissolved inorganic carbon (DIC) is involved.

The following equations describe photosynthetic carbohydrate formation from the various available CO_2_ species:

(6) \begin{eqnarray*} \displaystyle ({\mathrm{CO}}_{2}+{\mathrm{H}}_{2}\mathrm{O})\Leftrightarrow {\mathrm{CH}}_{2}\mathrm{O}+{\mathrm{O}}_{2}&&\displaystyle \end{eqnarray*}

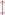

(7) \begin{eqnarray*} \displaystyle ({\mathrm{H}}^{+}+{\mathrm{HCO}}_{3}^{-})\Leftrightarrow {\mathrm{CH}}_{2}\mathrm{O}+{\mathrm{O}}_{2}&&\displaystyle \end{eqnarray*}

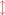

(8) \begin{eqnarray*} \displaystyle (2{\mathrm{H}}^{+}+{\mathrm{CO}}_{3}^{2-})\Leftrightarrow {\mathrm{CH}}_{2}\mathrm{O}+{\mathrm{O}}_{2}.&&\displaystyle \end{eqnarray*}

The photosynthesis equations are also written in two dimensions with the red arrows showing changes in distribution of species that occurs (Eq. (2)) with shifts in pH. Note that photosynthesis increases pH (lowers [H^+^]) while the reverse reaction of respiration decreases pH (increases [H^+^]). Net photosynthesis (*P*_net_) is the sum of photosynthesis (positive flux) and respiration (negative flux). Unlike calcification-dissolution, photosynthesis-respiration does not alter total alkalinity (*A_T_*).

In sum, photosynthesis and calcification both lower the seawater DIC, while respiration and CaCO_3_ dissolution raise DIC. Only the precipitation or dissolution of CaCO_3_ significantly alters *A_T_*. Consequently, changes in [*A_T_*] can be used to calculate calcification and dissolution rates (*G*_net_), and is widely used in this regard. Photosynthesis and respiration can radically alter [H^+^] and thus can alter relative concentration of CO_3_^2−^,HCO_3_^−^ and CO_2_. Coral calcification is a biological process that is heavily influenced by the associated processes of photosynthesis and respiration (*P*_net_) that modify pH. Protons can be considered a waste product of calcification Eqs. (3)–(5) and O_2_ a waste product of photosynthesis Eqs. (6)–(8).

## Methods and Materials

A mesocosm experiment was undertaken in order to precisely measure the changes in bulk sea water chemistry and material flux caused by coral and algae metabolism over a diurnal cycle. The experiment was conducted in the flow-through mesocosm system at the Hawaii Institute of Marine Biology, Kaneohe Bay, Oahu, Hawaii. The mesocosm system has been described previously in detail (Jokiel et al., 2008; Andersson et al., 2009; Jokiel, Bahr & Rodgers, in press). The fiberglass mesocosm tanks were located in full sunlight and supplied with flowing seawater pumped from approximately 2 m depth at the edge of the Coconut Island coral reef. Each mesocosm received a flow of approximately 7.5 to 8.5 l min^−1^ resulting in a turnover rate of approximately 1 h. Solar input at the site was monitored with a LiCor Brand Quantameter (Li-Cor Inc., Lincoln, NE, USA), which measured photosynthetically active radiance (PAR) between 400 nm and 700 nm.

Three continuous flow mesocosms were used for this experiment. The first mesocosm (“Coral only”) was loaded with 7.6 kg buoyant weight of the reef coral *Montipora capitata* for close to 100% coverage of the bottom (Table 1). This buoyant weight translates into 11.5 kg dry skeletal weight (Jokiel, Maragos & Franzisket, 1978). The second mesocosm (“Coral plus Algae”) contained the same weight of live coral plus 3.1 kg wet weight of the macroalgae *Gracillaria salicornia*. The third mesocosm (“Algae only”) was loaded with the same weight of the macroalgae. A small biomass of calcifying organisms was present on the macroalgae in the form of epiphytes that were not removed. Dead skeletal material and sediment were excluded from all three mesocosms to reduce the complicating effect of decalcification of carbonates and related processes on *G*_net_ (Murillo, Jokiel & Atkinson, 2014). The organisms in the mesocosms were allowed to acclimate to mesocosm conditions for one week prior to the experiment.

**Table 1:** Mesocosm volume and biomass of the coral *Montipora capitata* and the macroalga*Gracillaria salicornia*.

		Tank dimensions in cm			
Mesocosm	Biomass	Length	Width	Water depth	Volume (l)
Coral only	7,555 g buoyant weight coral	117	117	38	520
Coral plus Algae	7,555 g buoyant weight coral, and 3,151 g of *Gracillaria salicornia*	117	117	38	520
Algae	3,151 g of *Gracillaria salicornia*	117	117	35	479

This experimental design allows the three treatments to be run simultaneously which eliminates the between-treatment variance due to solar irradiance, temperature and other factors that would occur if they were run at different times. The functioning of each community under identical conditions serves as a control and as a contrast to the other two communities. Response of these communities to the diurnal irradiance cycle and variation in other factors is non-linear, so the data were analyzed by graphical comparison and integrated response of 24 h *P*_net_ and *G*_net_.

The seawater inflow enters at the bottom in the center of each mesocosm (see Smith et al., 1977 for details on the mesocosm system) at a vertical angle, which ensures a uniform and well-mixed system. Maintaining seawater inflow of reef water at constant rates insured that natural fluctuations in seawater chemistry observed on the adjacent reef during the diel cycle was preserved during the experiment. Water chemistry was sampled hourly from 06:00 on 24 April to 07:00 on 25 April 2012 at the inlet and outlet and the flow rate recorded. During the experiment the flow rate was precisely measured every hour for each mesocosm and inlet-outlet chemistry was determined. Temperature and salinity were measured with a YSI Brand Model 30 salinity–conductivity–temperature meter (±0.1 °C; ±0.1 ppt). Dissolved oxygen (DO) was measured with a YSI Brand Model 57 Dissolved Oxygen Meter (±0.2 mg l^−1^), and pH_NBS_ with an Accumet AP72 pH/mV/temperature meter verified spectrophotometrically using *m*-cresol purple dye according to SOP 7 (Dickson, Sabine & Christian, 2007). *A_T_* was measured using a Titrino Model 877 titrator system. Alkalinity samples were equilibrated to 25 °C and run within an hour of being taken. Accuracy and precision of the titrations was confirmed with certified reference materials (CRM Batch 129) from the Dickson Laboratory, Scripps Institution of Oceanography which verified that our measurements of *A_T_* were accurate to within 0.18% of the Dickson CRM value. All carbonate parameters were calculated using the program CO2SYS (Pierrot, Lewis & Wallace, 2006) and stoichiometric dissociation constants defined by Mehrbach et al. (1973) and refit by Dickson & Millero (1987).

The mesocosms are well mixed systems and can be represented by a simple box model Andersson et al. (2009). Changes in total alkalinity (*A_T_*) are attributed to calcification or carbonate dissolution Murillo, Jokiel & Atkinson (2014). The net calcification (*G*_net_) can be easily calculated from Eq. (9) by measuring the amount of material in the inflowing (*F*_in_) and outflowing (*F*_out_) seawater, and the change per unit time in the mesocosm seawater between consecutive sampling times. For *G*_net_ the resulting value is divided by 2 because two moles of *A_T_* are produced for every mole of CaCO_3_ precipitated (calcification) or removed (dissolution) as shown by Eqs. (3)–(5).

(9) \begin{eqnarray*} \displaystyle {G}_{\mathrm{net}}=\frac{{F}_{\mathrm{in}}{A}_{T}-{F}_{\mathrm{out}}{A}_{T}-\frac{d{A}_{T}}{d t}}{2}.&&\displaystyle \end{eqnarray*}

*P*_net_ was measured in a similar manner using O_2_ concentration, with one mole of carbon being produced for every mole of O_2_ produced as shown by Eqs. (6)–(8). Likewise DIC and H^+^ were calculated using Eqs. (11) and (12).

(10) \begin{eqnarray*} \displaystyle {P}_{\mathrm{net}}={F}_{\mathrm{in}}{\mathrm{O}}_{2}-{F}_{\mathrm{out}}{\mathrm{O}}_{2}-\frac{d{\mathrm{O}}_{2}}{d t}&&\displaystyle \end{eqnarray*}

(11) \begin{eqnarray*} \displaystyle {\mathrm{DIC}}_{\mathrm{net}}={F}_{\mathrm{in}}\mathrm{DIC}-{F}_{\mathrm{out}}\mathrm{DIC}-\frac{d\mathrm{DIC}}{d t}&&\displaystyle \end{eqnarray*}

(12) \begin{eqnarray*} \displaystyle {\mathrm{H}}_{\mathrm{net}}^{+}={F}_{\mathrm{in}}{\mathrm{H}}^{+}-{F}_{\mathrm{out}}{\mathrm{H}}^{+}-\frac{d{\mathrm{H}}^{+}}{d t}.&&\displaystyle \end{eqnarray*}

## Results and Discussion

All of the measurements taken during this experiment are shown in Table 2.

**Table 2:** Measurements made during the mesocosm experiment of April 24–25, 2012.

Time	Irradiance µ mole photons m^−2^ h^−1^	Salinity o/oo	Inlet—all mesocosms				Outlet Algae only mesocosm							
			pH	*A_T_*	Temp °C	O_2_ In mg l^−1^	Flow liters min^−1^	pH	A_*T*_	Temp °C	O_2_ Out mg l^−1^	*G*_net_	*P*_net_	Mean Ω_arag_
6:00	0	34.8	7.95	2196.5	23.1	6.0	7.50	7.93	2196.0	23.0	6.0			
7:00	56	34.9	7.95	2198.3	23.5	6.0	7.50	7.93	2196.7	23.2	6.1	0.3	22	2.49
8:00	222	34.8	8.03	2199.0	23.5	6.0	7.50	8.01	2193.5	23.3	6.4	1.0	127	2.70
9:00	464	34.9	8.03	2185.0	23.5	6.3	7.50	8.02	2190.7	23.4	7.1	0.0	340	2.93
10:00	854	34.8	8.06	2200.3	23.3	6.9	7.33	8.06	2182.9	23.6	8.0	1.6	539	3.09
11:00	1619	34.8	8.06	2206.2	23.9	6.6	7.50	8.11	2198.5	23.6	8.2	3.4	746	3.41
12:00	1705	34.8	8.09	2197.7	23.8	6.7	7.67	8.14	2199.6	24.4	7.8	0.8	756	3.70
13:00	1468	34.8	8.09	2200.1	24.0	6.6	7.50	8.16	2195.7	24.4	7.5	0.3	546	3.86
14:00	1702	34.8	8.10	2202.2	24.0	6.7	7.67	8.16	2194.7	24.6	7.8	1.6	534	3.93
15:00	1001	34.9	8.08	2194.6	24.1	6.7	7.83	8.15	2196.6	24.2	7.6	0.7	504	3.89
16:00	739	34.9	8.09	2208.4	24.1	6.6	7.67	8.15	2199.3	24.1	7.5	1.0	488	3.83
17:00	429	34.9	8.08	2208.1	24.0	6.4	7.67	8.12	2209.3	23.9	6.9	1.1	379	3.72
18:00	159	34.8	8.08	2210.7	23.9	6.3	7.67	8.08	2211.4	23.9	6.3	−0.3	127	3.49
19:00	32	34.9	8.07	2199.0	23.9	6.3	7.50	8.08	2203.3	23.5	6.2	−0.7	−50	3.34
20:00	0	34.9	8.07	2184.7	23.8	6.3	7.67	8.07	2189.4	23.5	6.0	−1.2	−135	3.28
21:00	0	34.9	8.07	2206.4	23.8	6.3	7.67	8.05	2203.0	23.5	6.0	−0.2	−179	3.18
22:00	0	34.9	8.04	2180.8	23.5	6.1	7.67	8.04	2199.1	23.3	6.0	−2.1	−147	3.10
23:00	0	34.9	8.01	2192.5	23.4	6.1	7.67	8.03	2199.4	23.3	5.9	−3.5	−87	3.05
0:00	0	34.9	8.06	2201.4	23.6	6.3	7.67	8.02	2199.2	23.3	6.0	−0.6	−124	2.99
1:00	0	35.0	8.04	2201.4	23.4	6.4	7.67	8.04	2201.2	23.2	5.8	0.3	−252	3.02
2:00	0	34.9	8.09	2214.8	23.7	6.3	7.67	8.06	2207.7	23.3	5.6	1.0	−344	3.13
3:00	0	34.9	8.06	2214.3	23.6	6.1	7.50	8.05	2207.0	23.1	5.8	2.0	−280	3.16
4:00	0	34.9	8.02	2209.2	23.3	6.2	7.50	8.04	2209.3	23.1	5.9	1.0	−169	3.10
5:00	0	34.9	8.10	2216.6	23.6	6.3	7.50	8.06	2211.6	23.4	5.8	0.7	−212	3.14
6:00	0	34.9	8.09	2214.3	23.5	6.1	7.50	8.07	2209.6	23.3	5.8	1.3	−216	3.23


Time	Outlet coral plus Algae mesocosm								Outlet coral only mesocosm							
	Flow liters min^−1^	pH	*A_T_*	Temp °C	O_2_ Out mg l^−1^	*G*_net_	*P*_net_	Mean Ω_arag_	Flow liters min^−1^	pH	*A_T_*	Temp °C	O_2_ Out mg l^−1^	*G*_net_	*P*_net_	Mean Ω_arag_
6:00	7.50	7.89	2190.8	23.0	5.6				8.33	7.93	2181.6	23.0	5.9			
7:00	7.50	7.92	2179.7	23.1	5.8	3.3	−264	2.36	8.33	7.89	2179.1	23.1	6.0	4.6	−59	2.38
8:00	7.50	7.97	2174.0	23.3	6.3	6.1	−67	2.55	8.00	7.96	2171.9	23.2	6.3	6.5	65	2.45
9:00	7.33	8.03	2165.2	23.4	7.5	6.2	361	2.83	8.17	7.99	2170.1	23.3	7.2	5.8	327	2.69
10:00	7.50	8.08	2156.9	23.5	8.9	8.5	883	3.12	8.33	8.04	2154.7	23.8	8.0	8.1	553	2.92
11:00	8.17	8.14	2165.3	24.3	8.4	11.6	956	3.49	8.17	8.10	2167.7	24.3	7.8	11.5	650	3.27
12:00	7.33	8.16	2165.7	24.5	8.4	10.2	983	3.80	8.00	8.12	2162.6	24.5	7.9	10.3	685	3.55
13:00	7.67	8.17	2162.9	24.4	8.1	9.3	853	3.89	8.33	8.13	2157.9	24.4	7.5	10.4	582	3.64
14:00	7.67	8.17	2159.3	24.6	8.4	10.8	845	3.93	8.33	8.12	2159.0	24.6	7.7	11.5	521	3.64
15:00	8.00	8.16	2164.4	24.3	8.1	9.5	768	3.89	8.67	8.10	2158.6	24.3	7.5	10.2	466	3.54
16:00	7.83	8.14	2174.3	24.0	7.8	9.0	644	3.77	8.00	8.09	2169.1	24.0	7.5	10.5	477	3.43
17:00	7.50	8.11	2183.0	23.9	7.0	8.3	472	3.61	8.00	8.09	2176.9	23.9	6.7	9.9	347	3.39
18:00	7.33	8.08	2194.5	23.6	6.2	5.6	61	3.43	8.33	8.06	2190.2	23.6	6.3	6.9	71	3.30
19:00	7.33	8.04	2190.3	23.6	5.9	3.4	−277	3.21	8.17	8.05	2187.2	23.5	6.0	4.4	−123	3.17
20:00	7.33	8.03	2172.5	23.2	5.9	2.9	−366	3.04	8.17	8.04	2177.7	23.3	6.0	2.6	−183	3.09
21:00	7.33	8.03	2192.6	23.2	5.6	3.6	−450	2.99	8.17	8.04	2192.5	23.2	5.8	2.9	−247	3.05
22:00	7.50	8.03	2189.4	23.2	5.6	0.7	−484	3.00	7.83	8.03	2185.6	23.3	5.7	1.3	−292	3.03
23:00	7.50	8.01	2190.4	23.1	5.6	−0.9	−410	2.94	8.17	8.02	2189.5	23.2	5.7	−0.2	−221	2.98
0:00	7.67	7.99	2188.3	23.2	5.6	2.0	−446	2.84	8.33	8.00	2188.6	23.2	5.8	2.1	−242	2.89
1:00	7.33	8.01	2190.2	23.2	5.4	3.3	−621	2.84	8.17	8.01	2190.2	23.2	5.6	3.3	−373	2.87
2:00	7.67	8.03	2196.5	23.3	5.2	4.0	−691	2.96	8.33	8.03	2194.1	23.2	5.4	4.3	−462	2.95
3:00	7.50	8.03	2197.2	23.2	5.3	4.9	−651	3.02	8.17	8.03	2195.7	23.2	5.6	5.4	−394	3.01
4:00	7.50	8.02	2200.2	23.1	5.4	3.6	−556	2.98	8.17	8.02	2199.3	23.1	5.7	3.9	−297	2.98
5:00	7.50	8.03	2200.5	23.4	5.4	3.4	−578	2.99	8.17	8.05	2199.1	23.2	5.6	3.8	−328	3.04
6:00	7.83	8.02	2200.3	23.3	5.4	4.0	−538	3.00	8.33	8.04	2199.1	23.2	5.7	4.4	−295	3.10

**Figure 1:**
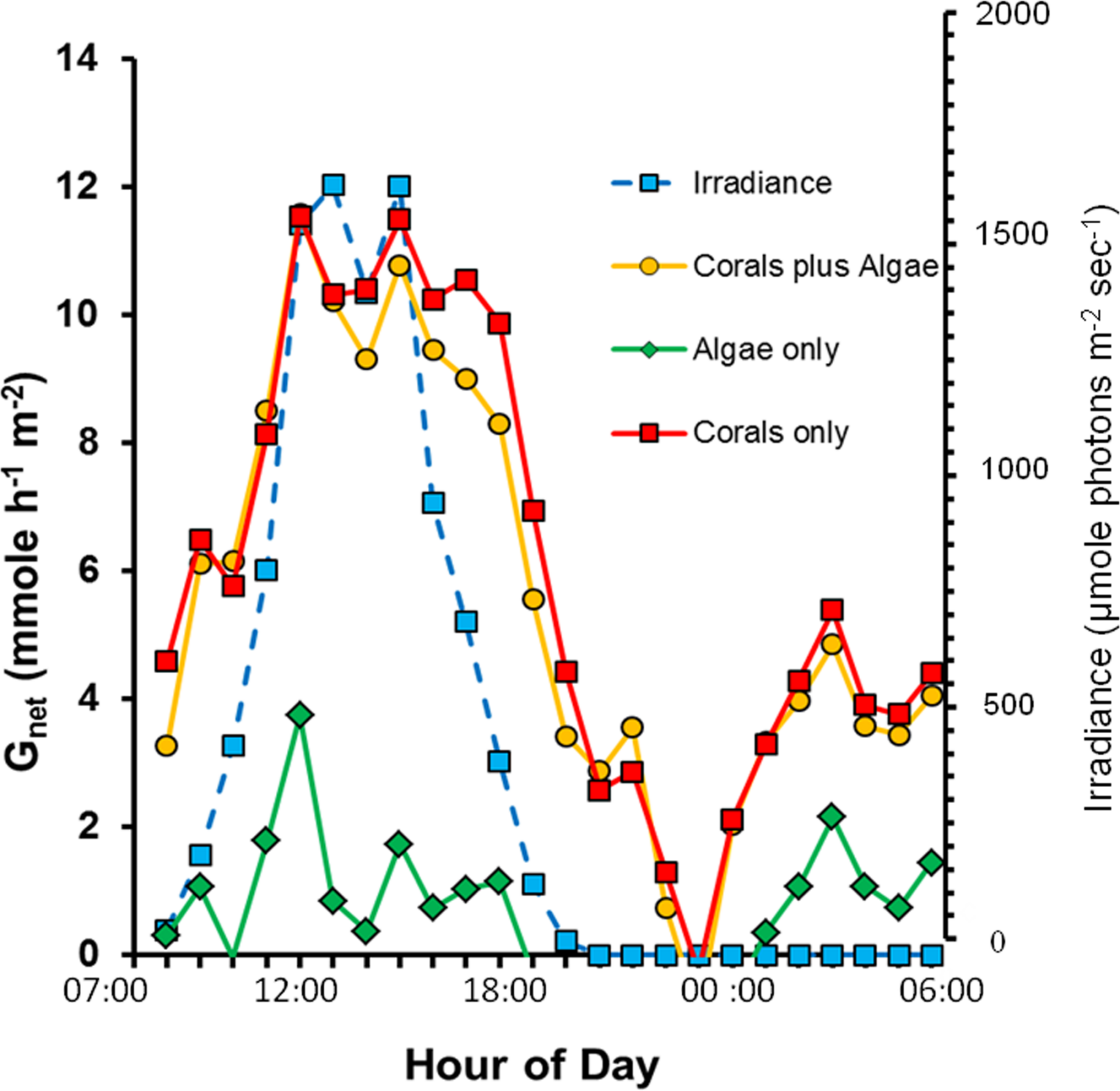
Diurnal net calcification rate (*G*_net_) and irradiance for the three mesocosms.

Calcification over the 24 h period (Fig. 1) shows the diurnal pattern related to irradiance, light enhanced calcification and dark calcification. Values for *G*_net_ are high due to the large biomass of live coral, high solar irradiance in the shallow mesocosms and absence of sediment or dead carbonate skeleton which are subject to dissolution. *G*_net_ in the “Corals only” and “Corals plus Algae” treatments track each other closely. Light saturation of calcification did not occur up to the maximum irradiance which exceeded 1,500 µmole photons m^−2^ s^−1^. This value is many times higher than that supplied by the artificial light typically used is most laboratory studies of coral calcification. Calcification rate is very low in the “Algae only” treatment due to low biomass of calcifying organisms, which are made up of various calcifying epiphytes. Low-levels of dark calcification occur at night. There is a drop in calcification to zero around midnight with a dark calcification rate peak at approximately 03:00 h.

### The relationship between *G*_net_ and Ω_arag_

**Figure 2:**
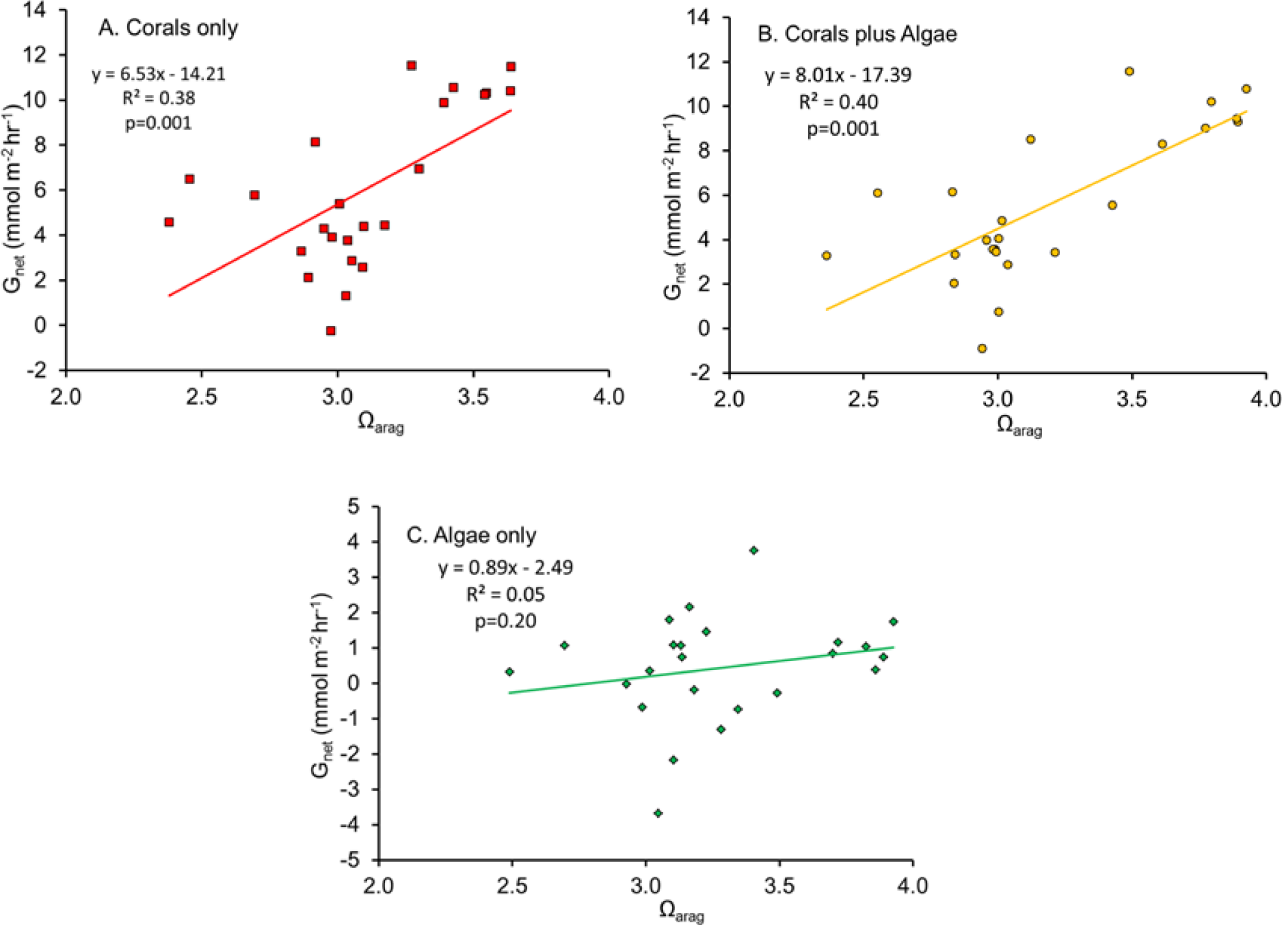
Net calcification rate (*G*_net_) plotted as a function of Ω_arag_ for (A) “Coral only” mesocosm, (B) “Coral and Algae” mesocosm and (C) “Algae only” mesocosm.

The linear regression of *G*_net_ plotted as a function of Ω_arag_ (Fig. 2) has become a widespread method of describing coral and coral reef calcification. A significant statistical relationship is obtained, with substantial variance that is generally assumed to be largely sampling error, or the result of other factors which influence *G*_net_. However, the explanation appears be more complex, as will be discussed in the following sections.

**Figure 3:**
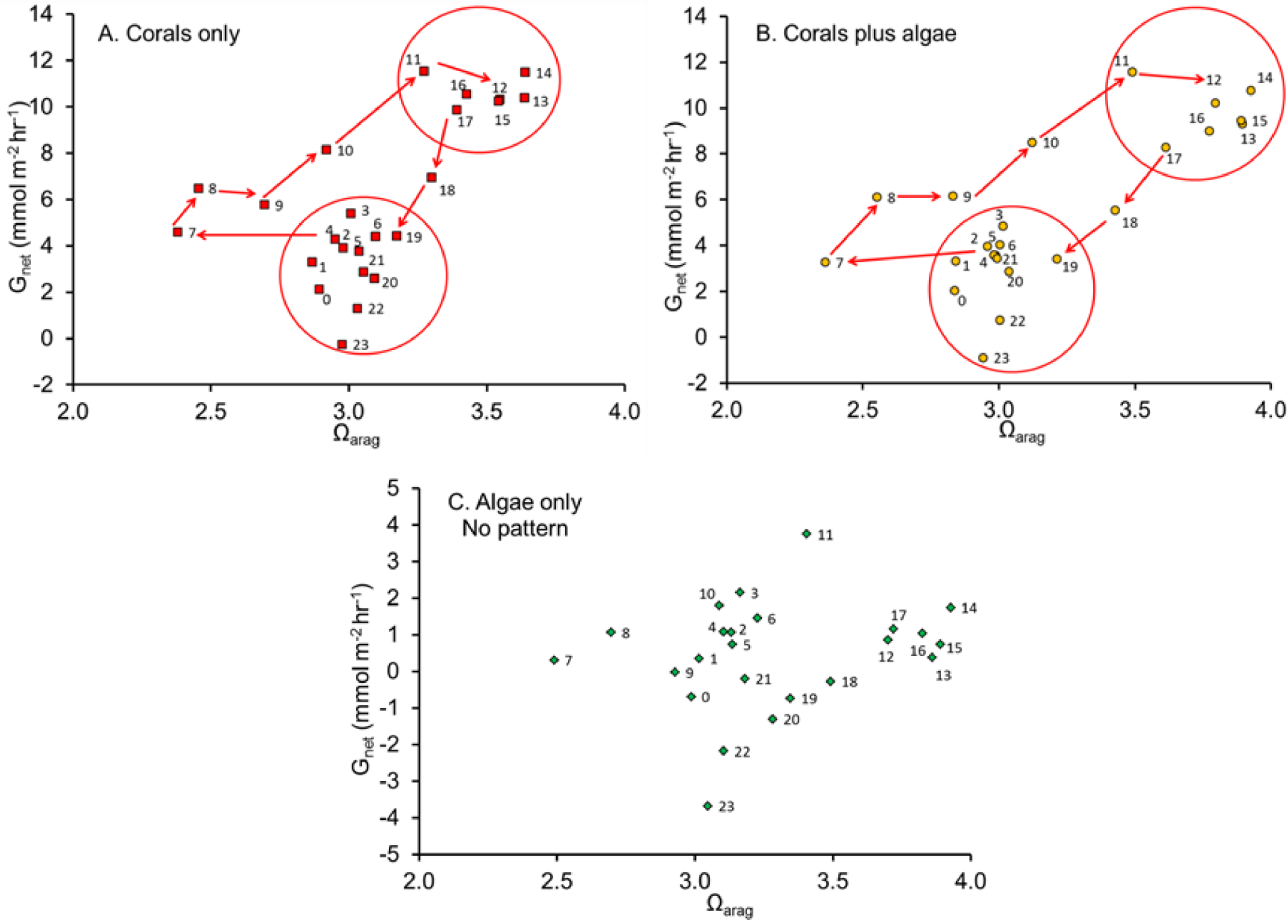
Net calcification rate versus Ω_arag_. Net calcification rate versus Ω_arag_ with each point labeled with hour of the day revealing the clockwise coral reef diel hysteresis pattern for: (A) “Coral only” mesocosm, (B) “Coral plus Algae” mesocosm and the (C) “Algae only” mesocosm, which showed no pattern.

### Diel hysteresis, phase lags and night calcification patterns

McMahon et al. (2013) quantified *G*_net_ in a healthy coral reef lagoon in the Great Barrier Reef during different times of day. Their observations revealed a diel hysteresis pattern in the *G*_net_ versus Ω_arag_ relationship. This phenomenon can be demonstrated by labeling the points in Fig. 2 with the hour of day as shown in Fig. 3. The diel pattern moves from the lower left quadrant early in the day toward the upper right through mid-day and then back to the lower center during the night before returning to the lower left quadrant at first light. The pattern is nearly identical for the “Corals only” mesocosm (Fig. 3A) and the “Corals plus Algae” (Fig. 3B), which tracked each other closely (Fig. 1). The “Algae only” mesocosm did not show this pattern. The linear regression for the *G*_net_ vs. Ω_arag_ data for the mesocosm with coral (Fig. 2) accounted for part of the variance (*R*^2^ = 0.40). A linear regression does not adequately describe the variance resulting from the diel pattern.

Cyronak et al. (2013) used chambers to measure *in situ* benthic solute fluxes at three different advection rates at Heron Island lagoon, Australia and observed a strong diurnal hysteresis pattern similar to that in Fig. 3. They suggested that diel hysteresis is caused by the diurnal interaction between photosynthesis and respiration. The data did not follow a trend consistent with the Ω_arag_ of the water column being the main driver of carbonate precipitation and dissolution. Instead, carbonate precipitation and dissolution in these sediment communities is linearly correlated to the rates of photosynthesis and respiration (*P*_net_) occurring over the same time period.

### Phase shifts

**Figure 4:**
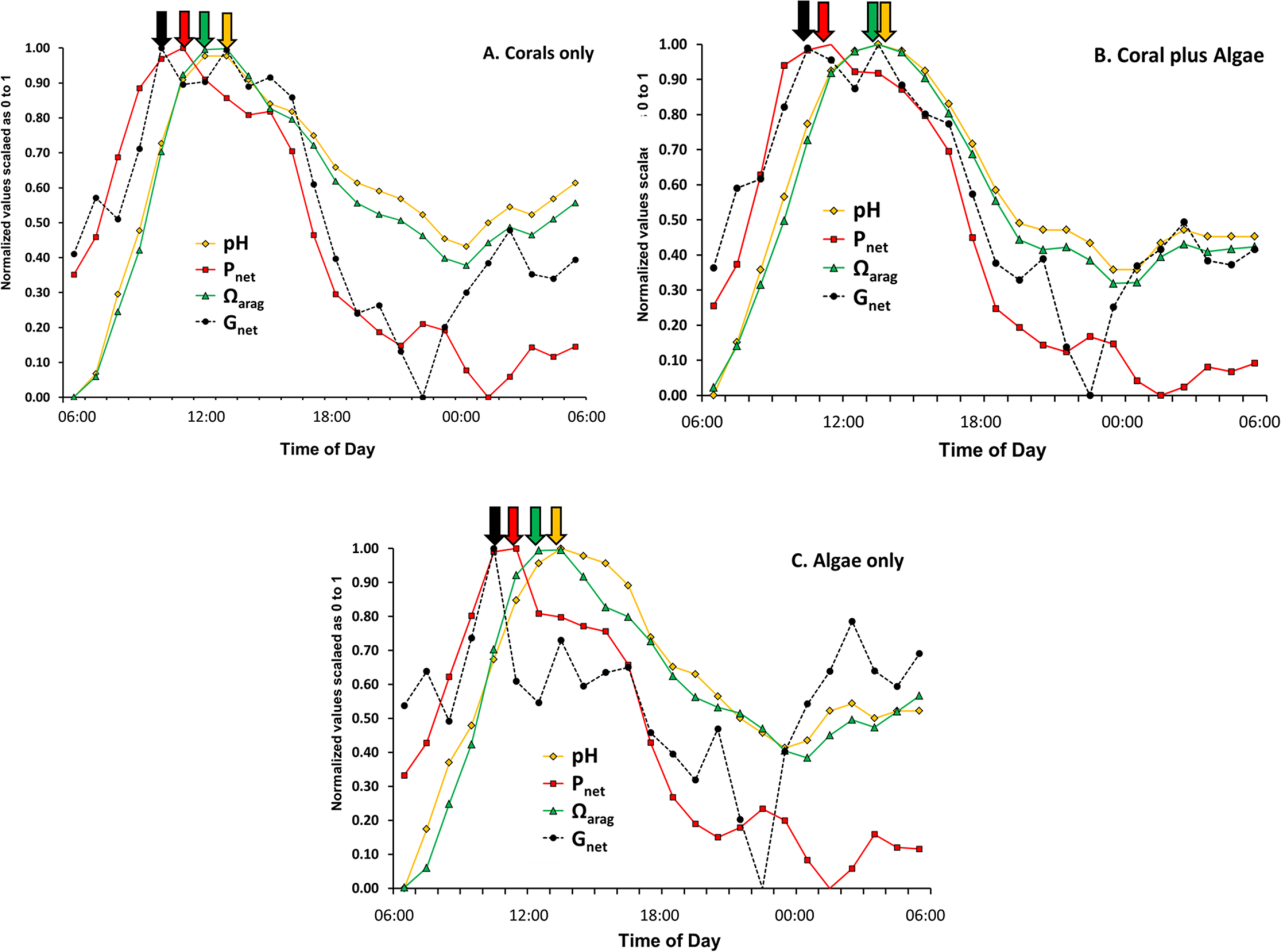
Normalized pH, Ω_arag_, *P*_net_ and *G*_net_ values for the three mesocosms versus time of day using data from Table 2 and Eq. (9). Arrows point to relative maxima for each parameter.

Evaluation of phase relations for the various parameters listed in Table 2 can be facilitated by scaling each variable on a 0 to 1 scale. The normalized value of *a_i_* for variable A in the *i*th row was calculated using the equation:

(13) \begin{eqnarray*} \displaystyle \text{Normalized value }({a}_{i})=\frac{{a}_{i}-{A}_{\mathrm{min}}}{{A}_{\mathrm{max}}-{A}_{\mathrm{min}}}&&\displaystyle \end{eqnarray*}

where *A*_min_ is the minimum value for variable *A* and *A*_max_ is the maximum value for variable. Figure 4 summarizes the results for the variables most often considered in the literature (pH, Ω_arag_, *P*_net_ and *G*_net_).

Figure 4 shows that peak pH and Ω_arag_ lag behind *G*_net_ throughout the daily cycle by two or more hours. The figure also shows that peak *G*_net_ follows *P*_net_ during daylight photosynthetic hours with a reverse during the nighttime hours. Shamberger et al. (2011) reported that Ω_arag_ lags behind *G*_net_ on the reefs of Kaneohe Bay, Hawaii. McMahon et al. (2013) reported that peak *G*_net_ rates occurred 2–3 h before the Ω_arag_ maximum on a healthy coral reef on the Great Barrier Reef. Thus Ω_arag_ (along with closely correlated [CO_3_^2−^], pH and [DIC]:[H^+^] ratio) is not the primary driver of coral calcification over a diurnal cycle. The paradigm that Ω_arag_ correlates with *G*_net_ on a global scale must be tempered with the caveat that other processes have a much greater influence on calcification on smaller spatial and temporal scales. The data presented above show that diurnal irradiance drives *P*_net_, which in turn drives *G*_net_, and which alters pH, which controls [CO_3_^2−^] and Ω_arag_ as well as other variables based on concentration such as the ratio of [DIC] to [H^+^]. A better understanding of this hierarchy will resolve many of the contradictions in the literature on coral reef calcification.

### Night calcification

**Figure 5:**
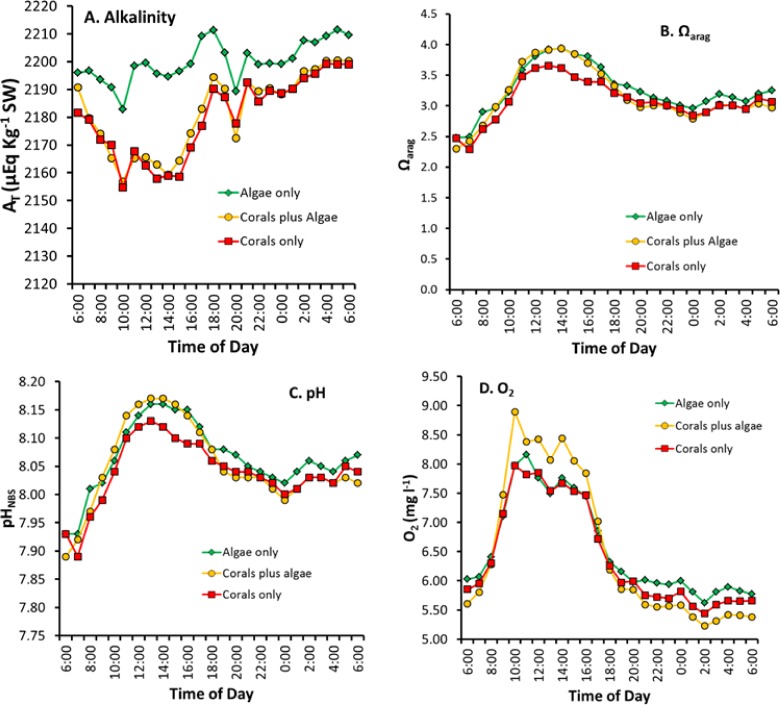
Diurnal changes in seawater chemistry in the three mesocosms for: (A) total alkalinity *A_T_*, (B) aragonite saturation state Ω_arag_, (C) pH and (D) dissolved oxygen.

Laboratory studies show that coral calcification continues in darkness, but at a lower rate than observed in light enhanced calcification (Schneider & Erez, 2006). Night calcification rates have generally been assumed to be low and constant at night, although this assumption has largely gone untested. Figures 1 and 4 show decreasing dark calcification following sunset, reaching zero near midnight followed by an increasing rate of dark calcification and an increase in respiration (Figs. 5C and 5D) that rises to a peak at 03:00 well before dawn. This pattern has occurred consistently in our mesocosm experiments, with the same pattern observed in 30 separate mesocosm runs with different communities under various conditions as well as in flume studies at our site (Murillo, Jokiel & Atkinson, 2014). Barnes & Crossland (1980) used time-lapse photography to measure diurnal growth in the staghorn coral *Acropora acuminata* and found that night-time extension rate was similar to or greater than day-time extension. They suggested that, “symbiotic association permits rapid growth because the coral can invest in flimsy scaffolding at night with the certainty that bricks and mortar will be available in the morning”. Wooldridge (2013) has proposed a new model for “dark” coral calcification, whereby O_2_-limitation of aerobic respiration during the night initiates a homeostatic host response that forms the skeletal organic matrix. The matrix formed at night subsequently allows rapid growth of the aragonite fibers during the “light-enhanced” period of calcification, when abundant energy derived from photosynthesis is available. Perhaps the mid-night calcification minimum observed in Figs. 1 and 4 at 00:00 reflects this period of organic matrix formation that precedes the 03:00 night calcification peak.

### Diurnal changes in concentration of *A_T_*, pH, Ω_arg_ and DO

The variables of *A_T_*, pH, Ω_arg_ DIC, and DO are concentrations while *P*_net_ and *G*_net_ are flux rates. Care must be taken when comparing concentrations to flux rates because flux rate can be high when concentration is high or low. Or flux rate can be low when concentration is high or low. Figure 4 shows patterns that are difficult to interpret because the figure mixes flux rates with concentrations. This issue will be discussed and resolved later in this discussion, but first we will compare differences in concentrations of key variables over the diurnal cycle (Fig. 5).

Figure 5 reveals several important patterns:

1. Alkalinity in the “Algae only” mesocosm remained high during the entire diurnal cycle. In contrast, the mesocosms containing corals showed lower *A_T_* (Fig. 5A) caused by rapid calcification. *A_T_* reduction by the corals was greatest during the daylight hours when *G*_net_ was high (Fig. 1) with the difference diminishing during nighttime hours.
2. The two mesocosms with algae maintained a higher Ω_arag_ throughout the mid-day portion of the diurnal cycle (Fig. 5B) which can be attributed to higher pH resulting from rapid rates of algae photosynthesis and coral photosynthesis (Eqs. (6)–(8)), with a less pronounced difference during the rest of the cycle.
3. The extreme difference in Ω_arag_ between the “Corals only” and the “Corals plus Algae” mesocosms (Fig. 5B) did not produce a corresponding difference in *G*_net_ between the two mesocosms (Fig. 1), which demonstrates that Ω_arag_ is uncoupled from *G*_net_ and explains differences encountered when comparing the Ω_arag_ versus *G*_net_ relationship in different systems with different diurnal *P*_net_ regimes.
4. Night-time pH and Ω_arag_ values (Figs. 5B and 5C) show less variability than *G*_net_ (Fig. 1).
5. The high biomass in the “Coral plus Algae” mesocosm (Fig. 5D) resulted in the highest O_2_ values during daylight hours (due to photosynthesis) and the lowest O_2_ during the night (due to respiration). The “Algae only” treatment had the second highest daytime level O_2_ due to algal photosynthesis and relatively high levels of O_2_ at night.
6. The 03:00 calcification peak observed in Fig. 1 is shown by both a decrease in O_2_ concentration and a drop in pH due to accelerated respiration. DO (Fig. 5D) and the pH (Fig. 5C) are measured independently and both show this effect to corroborate the observation.

**Figure 6:**
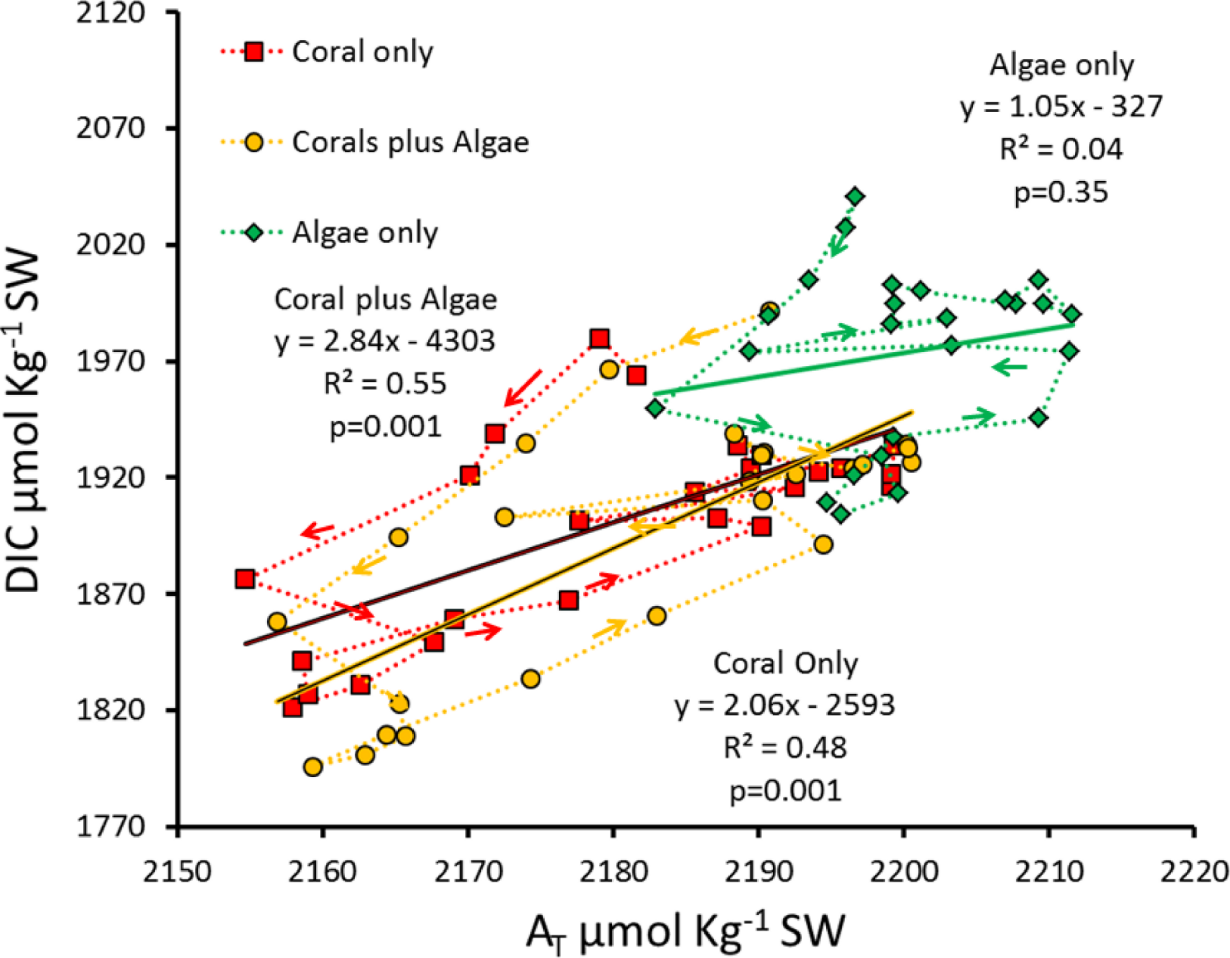
Hourly DIC versus *A_T_* for the diurnal data measured in each of the three mesocosms. Successive hours are connected by the dotted lines to show the patterns of diurnal hysteresis.

Plotting DIC versus *A_T_* (Fig. 6) demonstrates the major influence of *P*_net_ on seawater Ω_arag_. Calcification and dissolution shift *A_T_* values horizontally along the abscissa in Fig. 6 and influence DIC values vertically along the ordinate. However, photosynthesis and respiration change DIC along the ordinate without changing *A_T_*. The observed hysteresis pattern results from *P*_net_ driving *G*_net_ and increasing pH. A linear relationship accounting for only half of the variance (*R*^2^ ≈ 0.5) between DIC and *A_T_* was observed for the two rapidly calcifying mesocosms containing corals. This relationship does not hold for the low-calcification “Algae only” mesocosm.

As pointed out by McMahon et al. (2013), connecting the points on a graph of *A_T_* vs. DIC reveals a circular hysteresis pattern over the diel cycle as shown for the *G*_net_ versus Ω_arag_ plot (Fig. 6). *G*_net_ can account for changes in both the *A_T_* and DIC concentrations. However, *P*_net_ can only account for changes DIC concentration. Therefore, Ω_arag_ is a function of the changes in carbonate chemistry due to both *P*_net_ and *G*_net_, and any changes in DIC concentration relative to *A_T_* will result in different influences on Ω_arag_. For example, in systems with high organic production relative to calcification (Coral plus Algae mesocosm), Ω_arag_ will increase during daylight due to high pH caused by high uptake of CO_2_ used for photosynthesis (Fig. 5D). Conversely, in systems with low organic production relative to calcification (Coral only mesocosm), Ω_arag_ will decrease due to the uptake of *A_T_*. Any decrease in *G*_net_ associated with an increase in *P*_net_ will increase Ω_arag_ and change the way that *G*_net_ responds to OA. Therefore, any prediction of future global changes on coral reef *G*_net_ based on oceanic seawater Ω_arag_ must also take into account the influence of future localized *P*_net_ on *G*_net_ and Ω_arag_ as well as changes in carbonate dissolution of reef carbonates as described by Murillo, Jokiel & Atkinson (2014) for each reef location.

Comparisons between the hourly and daily *G*_net_ and *P*_net_ values (Table 3) show a similar calcification rate for both the “Coral only” mesocosm and the “Coral plus Algae” mesocosm in spite of the differences in DO, pH, *A_T_* (Fig. 5) and *P*_net_. However, daily *P*_net_ for the “Coral plus Algae” mesocosm was only one third of the *P*_net_ of the “Coral only” mesocosm. Hourly production was much higher in the “Corals plus Algae” mesocosm during the daylight hours, but production was consumed by extremely high respiration during nighttime hours. The “Algae only” mesocosm showed very low daily *G*_net_ and extremely high daily *P*_net_.

**Table 3:** Hourly and daily *G*_net_ and *P*_net_ values for the three mesocosms.

	Solar irradiance	*G*_net_ (mmol m^−2^ h^−1^)			*P*_net_ (mmol m^−2^ h^−1^)		
Time of day	µmol photons m^−2^ s^−1^	Corals only	Corals plus Algae	Algae only	Corals only	Corals plus Algae	Algae only
07:00	56.3	4.6	3.3	0.3	−59	−264	22
08:00	221.6	6.5	6.1	1.0	65	−67	127
09:00	464.6	5.8	6.2	0.0	327	361	340
10:00	854.0	8.1	8.5	1.6	553	883	539
11:00	1619.0	11.5	11.6	3.4	650	956	746
12:00	1705.0	10.3	10.2	0.8	685	983	756
13:00	1468.0	10.4	9.3	0.3	582	853	546
14:00	1702.0	11.5	10.8	1.6	521	845	534
15:00	1001.0	10.2	9.5	0.7	466	768	504
16:00	739.0	10.5	9.0	1.0	477	644	488
17:00	429.0	9.9	8.3	1.1	347	472	379
18:00	159.0	6.9	5.6	−0.3	71	61	127
19:00	31.7	4.4	3.4	−0.7	−123	−277	−50
20:00	0.0	2.6	2.9	−1.2	−183	−366	−135
21:00	0.0	2.9	3.6	−0.2	−247	−450	−179
22:00	0.0	1.3	0.7	−2.1	−292	−484	−147
23:00	0.0	−0.2	−0.9	−3.5	−221	−410	−87
24:00	0.0	2.1	2.0	−0.6	−242	−446	−124
01:00	0.0	3.3	3.3	0.3	−373	−621	−252
02:00	0.0	4.3	4.0	1.0	−462	−691	−344
03:00	0.0	5.4	4.9	2.0	−394	−651	−280
04:00	0.0	3.9	3.6	1.0	−297	−556	−169
05:00	0.0	3.8	3.4	0.7	−328	−578	−212
06:00	0.0	4.4	4.0	1.3	−295	−538	−216
Daily (mmolm^−2^d^−1^)		144	133	10	1226	427	2913

Anthony, Kleypas & Gattuso (2011) proposed a model that areas dominated by algal beds draw CO_2_ down and elevate Ω_arag_, potentially offsetting ocean acidification impacts at the local scale. Their model is based on the paradigm that *G*_net_ is controlled by Ω_arag_. Results suggested that a shift from coral to algal abundance under ocean acidification can lead to improved conditions for calcification (i.g. increased Ω_arag_) in downstream habitats and that alga beds can provide a significant mechanism for buffering ocean acidification impacts at the scale of habitat to reef. However, this conclusion is at odds with the measured values shown in Table 3 and Fig. 5. *G*_net_ in the “Corals plus Algae” treatment was the same as the “Coral Only” treatment even though the Ω_arag_ was much higher. In addition, the presence of the algae caused a precipitous drop in *P*_net_. The flaw in their model appears to be the assumption that *G*_net_ is controlled by Ω_arag_. Algal photosynthesis increases pH which shifts the equilibrium to higher [CO_3_^2−^] and thus higher Ω_arag_. There is a direct correlation between *G*_net_ and Ω_arag_ for a specific reef community, but not a cause and effect relationship.

### Diurnal changes in material flux (*P*_net_, G_net_, H^+^ flux and DIC flux)

**Figure 7:**
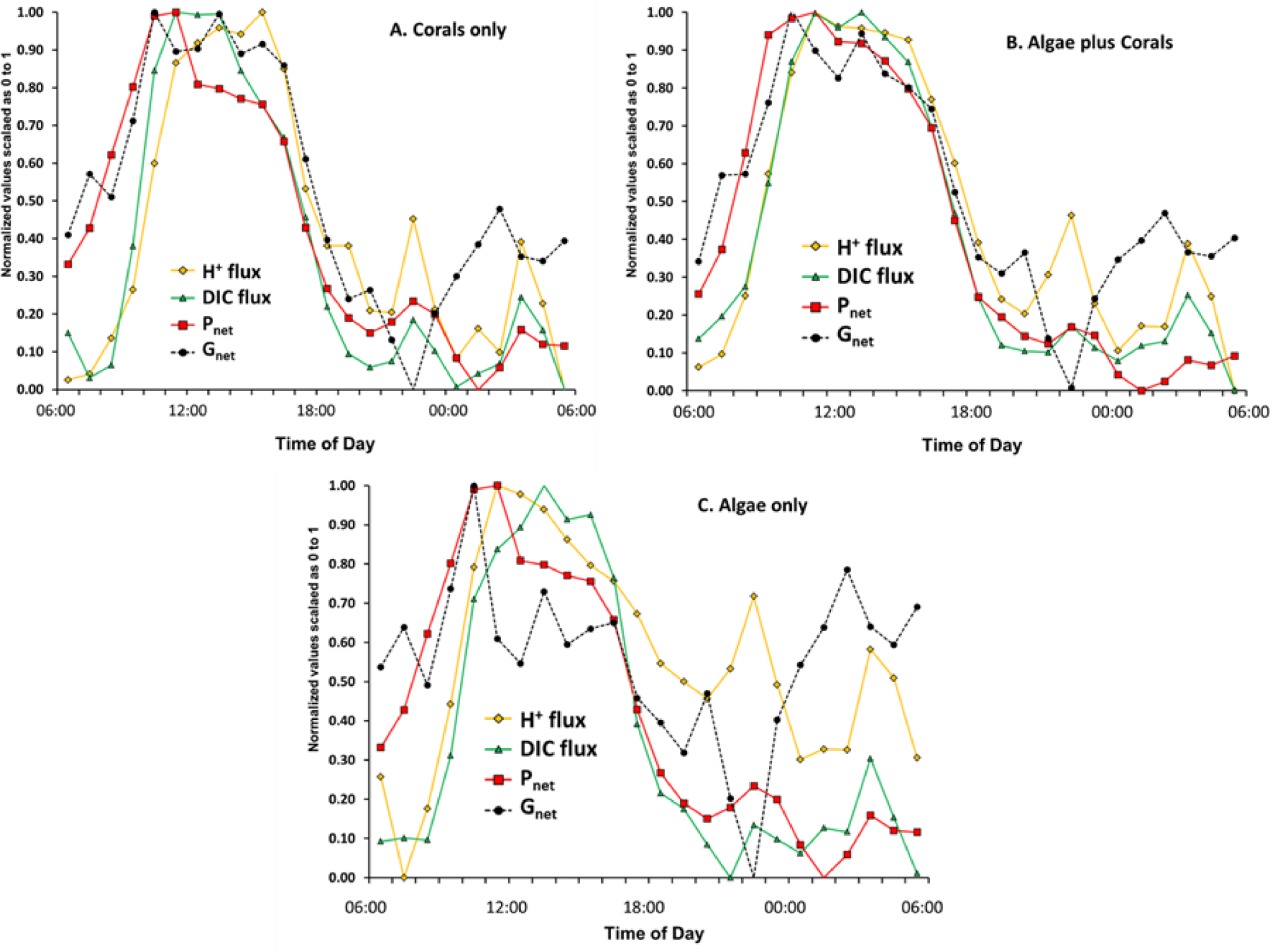
Plot of normalized data for *P_net_*, *G_net_*, inverse DIC flux and H^+^ flux for the experiment.

*G*_net_ and *P*_net_ are measures of material flux. DO, pH and DIC are measures of concentration. The preceding discussion and numerous publications often compare concentrations of one material to flux rate of another material or vice versa. Much more can be learned by plotting DIC flux and H^+^ flux rather than [DIC], [H^+^] or pH in relation to *P*_net_ and *G*_net_. DIC flux and H^+^ flux were calculated using the box model and graphed on a 0 to 1 scale in the same manner as in Fig. 4 with the result presented as Fig. 7. This figure illustrates the dynamic geochemical and physiological relationships involved in coral and coral reef metabolism.

DIC flux (uptake) in the highly calcifying mesocosms containing coral (Fig. 7) increases with increasing *P*_net_ from 06:00 until mid-day peak *P*_net_ and then decreases rapidly as *P*_net_ decreases with decreasing irradiance. Furla et al. (2000) demonstrated the presence of a DIC pool within coral tissues. The size of this pool was dependent on the lighting conditions, since it increased 39-fold after 3 h of illumination. If we apply this observation to the data shown in Fig. 7, it appears that the DIC pool had increased by mid-day, so rate of DIC uptake dropped rapidly as irradiance and photosynthesis declined. However, note that the high dissipation rates of H^+^ continued for 2–3 h following the peak rates of *P*_net_ and *G*_net_ as the corals rid themselves of the backlog of H^+^ generated by rapid calcification. Thus the lag of pH behind the peak flux rates of *P*_net_ and *G*_net_ (Figs. 4A and 4B) represents a disequilibrium that results from the lag in proton efflux from the corals. The correlation between Ω_arag_ and *G*_net_ is simply the response of the CO_2_-carbonate system to pH as [H^+^] shifts the equilibria and redistributes the [CO_3_^2−^] relative to the other DIC components of [HCO_3_^−^] and [CO_2_] (Eqs. (3)–(5)) . Therefore Ω_arag_ closely tracks pH whereas *G*_net_ tracks *P*_net_ more closely. Changes in Ω_arag_ are a consequence of changes in *P*_net_ and *G*_net_, rather than a driver of *G*_net_. Hence the Ω_arag_ peak and the pH peak lag behind the *P*_net_ and *G*_net_ peaks (Figs. 4A and 4B) due to lag in proton efflux seen in Fig. 7. This observation demonstrates the importance of understanding the difference between H^+^ concentration and H^+^ flux.

During the night the H^+^ flux rate is very responsive to changes in *G*_net_ in the “Algae only” and “Coral plus Algae” due to large changes in respiration (Fig. 7). The fluctuations of proton flux at night in the “Coral only” mesocosm are dampened considerably compared to the “Algae only” treatment. The “Coral plus Algae” mesocosm shows an intermediate response. Perhaps the coral skeleton acts as a buffer in a manner similar to that proposed by Suzuki, Nakamori & Kayanne (1995). The macroalgae lack the large skeletal carbonate buffer of reef corals.

### Back to the basics

**Figure 8:**
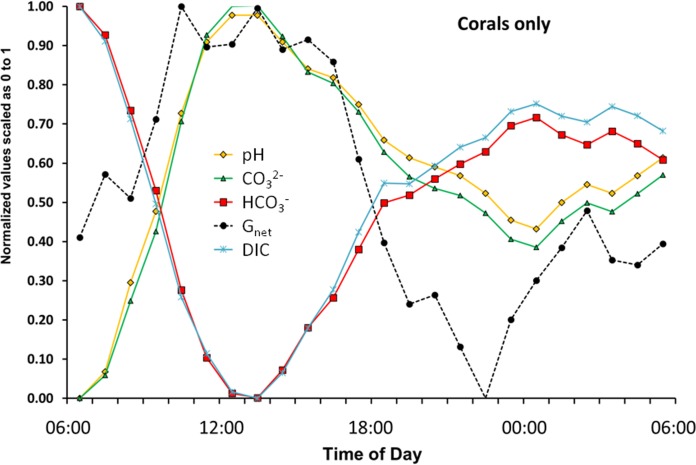
The flux rate of calcification-dissolution (*G*_net_) plotted against the concentrations of important variables of the CO_2_-carbonate system for the “Corals only” mesocosm.

The preceding sections have established the importance of using flux rates rather than concentrations when we are describing a dynamic metabolic system such as a coral or coral reef. Most of the previous research in this area has focused on the relationship between *G*_net_, [CO_3_^2−^] (or its surrogate Ω_arag_), [HCO_3_^−^], and [H^+^] expressed as pH. Plotting these variables in exemplary Fig. 8 is very informative and sheds light on results of previous studies.

A coral must uptake inorganic carbon in order to maintain photosynthesis and calcification. As a result [DIC] will decrease no matter which carbonate species (HCO_3_^−^, CO_3_^2−^ or CO_2_) is taken up by the coral (Eqs. (3)–(5)). Thus we see a decline in [DIC] at high rates of *G*_net_ (Fig. 8). [HCO_3_^−^], which has been identified as the preferred substrate for photosynthesis and calcification (Weis, Smith & Muscatine, 1989; Furla et al., 2000; Roleda, Boyd & Hurd, 2012) closely tracks [DIC] during daylight hours. In contrast, [CO_3_^2−^] lags behind *G*_net_ and closely tracks pH during the day as shown for Ω_arag_ in Fig. 4. If [CO_3_^2−^] (or its surrogate Ω_arag_) drives calcification, then how do we explain the lag behind *G*_net_? And if [CO_3_^2−^] is limiting, how do we explain the fact that [CO_3_^2−^] is increasing rather than decreasing as the coral calcifies rapidly and takes up inorganic carbon? [CO_3_^2−^] increases because of the increase in pH caused by rapid photosynthesis, which shifts the equilibrium between [HCO_3_^−^] and [CO_3_^2−^]. Thus, *P*_net_ is the driver of changes in *G*_net_ and [CO_3_^2−^] (Eqs. (2)–(5)). A basic physiological interpretation of the patterns shown in Fig. 8 is that daytime coral metabolism rapidly removes DIC (primarily in the form of HCO_3_^−^) while photosynthesis provides the energy that drives *G*_net_ (Fig. 4). Higher pH resulting from rapid photosynthesis pushes the equilibria toward higher [CO_3_^2−^]. This scenario results in a correlation between *G*_net_ and Ω_arag_, with Ω_arag_ as the dependent variable.

During the night [HCO_3_^−^], [DIC], [CO_3_^2−^] and pH mirror changes in *G*_net_. However, note that [HCO_3_^−^] diverges from [DIC] and [CO_3_^2−^] diverges from pH in darkness. The night divergence can be attributed to respiration causing a decrease in pH. The decreasing pH shifts the equilibria so that [CO_3_^2−^] is converted to [HCO_3_^−^], thereby changing the offset between the points. This phenomenon is also reflected in the pattern of diurnal hysteresis show in Fig. 3.

## Conclusions and Recommendations

### Correlations do not establish cause and effect

Linear regression using Ω_arag_ as the independent variable may be useful as a first approximation, but is a poor descriptor of calcification dynamics on coral reefs. Much of the existing data on coral calcification was developed in static or low turnover incubation experiments under typical laboratory low irradiance artificial light sources on a 12 h light, 12 h dark cycle (Jokiel, Bahr & Rodgers, in press). This regime results in an unrealistic simulation of the actual diurnal cycle that occurs on coral reefs. The standard protocol has been to compare linear regressions between or among treatments. Linear regression provides a very limited description of the actual relationship between the key factors controlling organic and inorganic processes on coral reefs, which are more adequately described by data presentations such as that in Table 3 or Fig. 7. The linear regression approach does not fully embrace natural diurnal calcification patterns and phase lags because these processes are non-linear. The linear regression approach can lead to the assumption that Ω_arag_ is the independent variable driving the calcification reaction. Nevertheless, correlations at a single location or in a single experiment can result where all other factors are held constant because the two quantities are related to some extent. Use of Ω_arag_ as an independent variable to compare spatial and temporal variation in *G*_net_ is known to create difficulties (Shamberger et al., 2011; Falter et al., 2012).

Numerous field and laboratory studies have demonstrated a positive correlation between *G*_net_ and Ω_arag_ for corals and coral reefs Erez et al. (2011). Well-developed reefs occur within a narrow geographic range characterized by open ocean Ω_arag_ > 3.3 (Kleypas et al., 1999), which could mean that coral communities have limited capacity to adapt to future levels of anthropomorphic ocean acidification (OA) projected for the 21st century. Recent reports suggest that healthy coral reefs could cease to exist within this time frame as OA continues and oceanic Ω_arag_ decreases (Hoegh-Guldberg et al., 2007; Silverman et al., 2009). However, there are inconsistencies in the relationship (slope and *x*-intercept) between *G*_net_ as a function of Ω_arag_ on various reefs throughout the world (Shamberger et al., 2011). For example, Kaneohe Bay, Oahu, Hawaii contains rich coral reefs that show extremely high rates of *G*_net_ while living at low Ω_arag_ levels (mean Ω_arag_ = 2.85) (Shamberger et al., 2011). Shamberger et al. (2014) report the existence of highly diverse, coral-dominated reef communities at the Rock Islands of Palau that are living at low saturation states (Ω_arag_ = 1.9–2.5). These values approach those projected for the tropical western Pacific open ocean by 2100 under future OA modeling scenarios. Identification of biological and environmental factors that enable these communities to persist at low Ω_arag_ could provide important insights into the future of coral reefs under increasing OA. So how do we account for the paradox of rich coral reefs growing at low Ω_arag_? Previous work has been based on the assumption that *G*_net_ is controlled by or related directly to Ω_arag_. The present investigation indicates that *P*_net_ rather than Ω_arag_ drives *G*_net_ and that calcification rate is further limited by proton flux, with pH and *A_T_* playing the major role in controlling calcification. Ω_arag_ on coral reefs is simply a dependent variable being controlled largely by changes in pH due to photosynthesis. A correlation will exist at a given site, but will not be consistent between different coral reef communities.

The [DIC]:[H^+^] ratio correlates with Ω_arag_ in describing *G*_net_ and is useful from a physiological point of view because it involves pH and all of the inorganic carbon species (Jokiel, 2011a; Jokiel, 2013). However, the [DIC]:[H^+^] ratio is simply another variable based on concentration (along with pH, Ω_arag_, CO_3_^2−^, etc.) that shows a correlation with *G*_net_. Nevertheless, the [DIC]:[H^+^] ratio can be important in describing *G*_net_ in situations where Ω_arag_ is decoupled from [H^+^] as occurs in the paleo-ocean over time scales greater than 10,000 years (Hönisch et al., 2012). A calcifying organism must uptake DIC in order to continue the calcification reaction and must rid itself of the waste protons. So *G*_net_ correlates directly to [DIC] and inversely to [H^+^].

Results of this investigation (Fig. 7) demonstrate the difficulty that corals encounter in shedding waste protons generated during calcification. High rates of H^+^flux continued for several hours following peak *G*_net_. An important conclusion of this work is that measurements of DIC flux and H^+^ flux are far more useful in describing coral metabolism dynamics than [DIC] and [H^+^] (Fig. 8). Likewise, Ω_arag_ is not a very useful variable in that it simply tracks pH (Fig. 4). This pattern becomes clear when one considers that [CO_3_^2−^] (and hence Ω_arag_) shifts with changing [H^+^] as described in Eqs. (2)–(5). DIC flux follows *P*_net_ and *G*_net_ and decreases rapidly following peak *P*_net_ and peak *G*_net_ indicating that corals can cope more effectively with the problem of DIC supply compared to the problem of eliminating H^+^.

### Future research directions

The time lag between *G*_net_ and Ω_arag_ reported previously in field studies (Shamberger et al., 2011; Cyronak et al., 2013; McMahon et al., 2013) provides evidence that diffusion and advection of materials between the coral and the water column involves time delays. One reason is that corals convert inorganic carbon to organic carbon, translocate the organic carbon to distal calcification sites, store organic carbon as lipid, and can eventually convert stored organic carbon back to inorganic carbon (Jokiel, 2011b), creating numerous possible phase lags for metabolic materials. The second reason for the time lag is that rapidly calcifying systems have difficulty dissipating waste protons as shown by continued rapid proton efflux for hours after peak calcification (Fig. 7). What other mechanisms can account for the phase lag? Boundary layers (BL) can slow the exchange of metabolic materials between the coral and the water column. The results of Cyronak et al. (2013) revealed that stirring had a net stimulatory effect on *A_T_* flux and on the diurnal cycle of hysteresis. Boundary layers slow exchange of metabolic materials, so this is an area of investigation that can provide an explanation.

Three hydrodynamic boundary layers have previously been defined and measured (Shashar, Cohen & Loya, 1993; Shashar et al., 1996). The Diffusion Boundary Layer (DBL) is only a few mm thick and in contact with the coral epidermis. The Momentum Boundary Layer (MBL) controls water movement in the proximity of the sessile organisms and is thicker by an order of magnitude than the DBL. The Benthic Boundary Layer (BBL), which controls the interactions of the reef with the surrounding sea water, was typically found to be more than 1 m thick and characterized by a roughness height of 31 cm and a shear velocity of 0.42 cm s^−1^ in the studies.

The DBL is a thin layer of stagnant seawater adjacent to the coral produced by frictional drag. This quiescent layer influences the flux of material between the benthic surface and the water column. The transport of Ca^2+^, CO_2_, CO_3_^2−^, HCO_3_^−^, O_2_, nutrients and H^+^ through the DBL is limited by the physical processes of diffusion and advection (Jokiel, 1978; Lesser et al., 1994; Kaandorp et al., 2005; Kaandorp, Filatov & Chindapol, 2011). Kühl et al. (1995) found that zooxanthellae photosynthesis resulted in a build-up of O_2_ in the photosynthetic tissue of up to 250% saturation and a tissue pH of up to 8.6 (i.e., 0.7 pH units above the pH value of the overlying seawater). In darkness the O_2_ within the coral tissue was depleted by respiration to near anoxic (<2% air saturation) conditions, with tissue pH of 7.3–7.4. O_2_ and pH profiles demonstrated the presence of a 200–300 µm thick DBL that separated the coral tissue from the overlying flowing seawater. Various models invoke boundary layer controls on coral metabolism. Kaandorp et al. (2005) and Kaandorp, Filatov & Chindapol (2011) addressed DBL limitation of DIC influx while Jokiel (2011a), Jokiel (2011b) and Jokiel (2013) challenged the paradigm that calcification is limited by CO_3_^2−^ supply on the reactant side of the calcification equation. Rather, he argued that rate of dissipation of H^+^ on the product side due to boundary layer conditions can be the actual limiting factor.

Boundary layer limitation of photosynthesis provides an analog to boundary layer limitation of calcification. Photosynthetic rate can be limited by rate of waste O_2_ dissipation through the boundary layer rather than being limited by supply of reactant CO_2_. By analogy, calcification can be limited by rate of removal of waste protons rather than by availability of inorganic carbon. The importance of water motion in reducing boundary layer thickness and thereby increasing oxygen flux between the photosynthetic organisms and the water column has been demonstrated (Mass et al., 2010). By analogy, increased water motion can decrease boundary layer thickness and thereby increase removal of protons from the coral.

Studies of reef metabolism beginning with the classic work of Odum & Odum (1955) at Enewetak Reef flat and followed by others (Shamberger et al., 2011; Falter et al., 2012) were conducted in shallow water reef flats within the BBL in situations where unidirectional currents allowed calculation of flux rates. Substantial boundary layers occur over all reefs. For example, Price et al. (2012) investigated a range of sites from exposed coastal situations to lagoons and found that ambient variability in pH was substantial and oscillated over a diurnal cycle with diel fluctuations in pH exceeding 0.2. Daily pH maxima were identified as an important control on calcification. Net accretion among sites was positively related to the magnitude and duration of pH above the climatological seasonal low, despite myriad other ecological (e.g., local supply, species interactions, etc.) and physical oceanographic (e.g., temperature, current magnitude and direction, wave strength, latitudinal gradients, etc.) drivers. In general, accretion rates were higher at sites that experienced a greater number of hours at high pH values each day. Where daily pH within the BBL failed to exceed pelagic climatological seasonal lows, net accretion was slower and fleshy, non-calcifying benthic organisms dominated space. Thus, key aspects of coral reef ecosystem structure and function are clearly related to natural diurnal variability in pH, which is driven primarily by photosynthesis and respiration as *P*_net_.

### The master variables

The practice of calculating and comparing linear regressions of *G*_net_ vs. Ω_arag_ to obtain a first approximation of calcification rates under different conditions is fraught with problems but probably will continue because it is ingrained in science and is convenient to use. The correlation of a primary biological response (*G*_net_) to a primary physical chemistry measurement (Ω_arag_) is attractive, especially in modeling the possible future changes on coral reefs. Unfortunately, the physical chemistry concept of Ω_arag_ has no basic physiological meaning in describing *G*_net_ other than a correlation with the [DIC]: [H^+^] ratio (Jokiel, 2013) as well as with other factors such as pH. There is no consistent relationship between Ω_arag_ and *G*_net_ when comparing reefs throughout the world (Shamberger et al., 2011). Coral reefs are systems in constant disequilibrium with the water column. So we must take care not to be led astray in our thinking about the variables that actually drive and control coral and coral reef metabolism and bulk water chemistry. The correlation between *G*_net_ and other factors is a result of *P*_net_ driving both *G*_net_ and Ω_arag_ (McMahon et al., 2013). The observed phenomenon of diurnal hysteresis and diurnal phase lag show the importance of measuring flux rates and emphasizes the challenge in predicting the future effects of OA on coral reefs. The method of using linear extrapolations of Ω_arag_ to determine threshold levels that will shift coral reefs from net calcifying systems to a net dissolving state has been questioned (McMahon et al., 2013). Perhaps predicted changes in Ω_arag_ in the open ocean can be used to calculate changes on reefs if we assume that the baseline on the reefs will change in concert with ocean values and that all other processes such as *P*_net_ and carbonate dissolution will not be influenced by OA. An explanation for the many paradoxes of coral calcification discussed herein has been presented as the “Two Compartment Proton Flux Model of Coral Metabolism” (Jokiel, 2011b). This model is focused on localized gradients that influence coral metabolism with a focus on proton flux, carbon pools and translocation of fixed carbon. A major feature of the model is the presence of boundary layers which control local pH gradients and inorganic carbon speciation in addition to proton flux. Results of the present investigation support this model.
